# INDs for PET Molecular Imaging Probes—Approach by an Academic Institution

**DOI:** 10.1007/s11307-014-0735-2

**Published:** 2014-04-15

**Authors:** Sherly Mosessian, Sandra M. Duarte-Vogel, David B. Stout, Kenneth P. Roos, Gregory W. Lawson, Maria C. Jordan, Amanda Ogden, Cheryl Matter, Saman Sadeghi, George Q. Mills, Heinrich R. Schelbert, Caius G. Radu, Johannes Czernin, Marcelo Couto, Michael E. Phelps

**Affiliations:** 1 Department of Molecular and Medical Pharmacology, David Geffen School of Medicine at University of California Los Angeles, 650 Charles E. Young Dr. South, CHS 23-148, Los Angeles, CA 90095 USA; 2 Division of Laboratory Animal Medicine, David Geffen School of Medicine at University of California Los Angeles, Los Angeles, CA USA; 3 Crump Institute for Molecular Imaging, David Geffen School of Medicine at University of California Los Angeles, Los Angeles, CA USA; 4 Department of Physiology, David Geffen School of Medicine at University of California Los Angeles, Los Angeles, CA USA; 5 Ahmanson Translational Imaging Division, David Geffen School of Medicine at University of California Los Angeles, Los Angeles, CA USA; 6 Perceptive Informatics/PAREXEL, Gaithersburg, MD USA

**Keywords:** PET probes, Investigational New Drug application, FDA regulations, Cost-effective, Clinical trial

## Abstract

We have developed an efficient, streamlined, cost-effective approach to obtain Investigational New Drug (IND) approvals from the Food and Drug Administration (FDA) for positron emission tomography (PET) imaging probes (while the FDA uses the terminology PET drugs, we are using “PET imaging probes,” “PET probes,” or “probes” as the descriptive terms). The required application and supporting data for the INDs were collected in a collaborative effort involving appropriate scientific disciplines. This path to INDs was successfully used to translate three [^18^ F]fluoro-arabinofuranosylcytosine (FAC) analog PET probes to phase 1 clinical trials. In doing this, a mechanism has been established to fulfill the FDA regulatory requirements for translating promising PET imaging probes from preclinical research into human clinical trials in an efficient and cost-effective manner.

**Electronic supplementary material:**

The online version of this article (doi:10.1007/s11307-014-0735-2) contains supplementary material, which is available to authorized users.

## Introduction

positron emission tomography (PET) is an exceptionally sensitive and noninvasive imaging modality that produces a quantitative, three-dimensional image of biological and pharmacological processes occurring in the body. This study’s approach involves administration of PET imaging probes and detection of the resulting 511-MeV gamma rays, due to positron decay, with a PET scanner. Over the past three decades, more than 2,000 PET probes have been developed [1] to provide assays of a wide array of biological and pharmacological processes. Based on the data available from the National Institutes of Health, Molecular Imaging and Contrast Agent Database, 1,361 PET probes have been utilized in animal studies and only 118 of these PET probes to date have been used in human studies. This rather low-percentage transition of probes to investigations in humans can be attributed to (1) nonoptimal bio-distribution and *in vivo* targeting of probes in animal models, understanding that there are limitations in the translation of animal bio-distribution data to humans, and (2) the difficulties and costs associated with gathering data and filing appropriate applications for Food and Drug Administration (FDA) approval to initiate these studies in humans. Thus, it is reasonable to surmise that there is a critical but unmet need to identify an efficient and cost-effective methodology of translating promising PET probes from the preclinical to the clinical domain and obtaining first-in-human data.

**Fig. 1.**
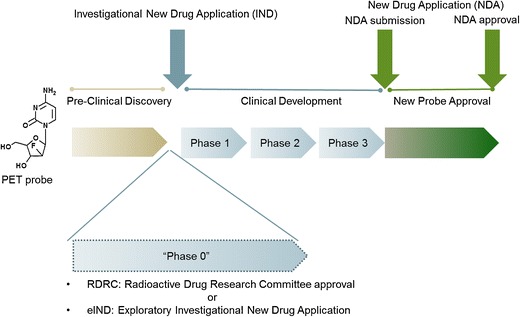
FDA approval process for PET imaging probes. Following the preclinical R&D discovery phase, PET probes enter the phases of clinical development through the IND application pathway. In addition to the IND pathway, when specific criteria are met, PET probes can be introduced into human subjects as part of the early clinic phase (phase 0) using the RDRC or eIND mechanisms. It is important to note the RDRC mechanism is not appropriate for first-in-human PET studies, and the detailed requirements for use of the RDRC mechanism are discussed in the text. An IND application is ultimately required for clinical development of the PET probe past phase 0. FDA reviews the sufficiency of human safety and efficacy data obtained during the phases of clinical development, and an NDA application is submitted by the sponsor to seek marketing approval. The PET probe is considered approved once the FDA completes the review and pre-approval inspection of the manufacturing site and considers the application submitted satisfactory for approval.

Approval for administration of the PET probes to human subjects is regulated by the FDA [2]. As directed by Section 121 (c)(1)(A) Modernization Act of 1997, the FDA has developed specific current good manufacturing practice (cGMP) requirements for manufacturing, quality control testing, and application submission for the approval of PET probes for human use [3–5]. Currently, there are several paths available for research use of PET imaging probes in human subjects (Fig. 1 and Table 1):

**Table 1:** Comparison of RDRC, eIND, and IND approaches for PET probes

	RDRC	eIND	IND
Purpose	-Only for basic research, where the pharmacologic dose of the drug to be administered is known not to cause any detectable pharmacologic effect in humans	-Only for basic research	-For clinical investigation of radiolabeled probes
		-Can be used to screen 2–5 probes simultaneously	
			-For therapeutic, diagnostic and preventative use
		-For micro-dose studies	
			-For determining safety and efficacy
		-When completed, must withdraw and transition to an IND	
	-Not intended for diagnostic/therapeutic		
		-Not intended for diagnostic or therapeutic	
Requirements	-Clinical protocol	-Clinical protocol	-Clinical protocol
	-Manufacturing under USP 823 or CFR 212 guidelines	-Manufacturing under USP 823 or CFR 212 guidelines	-Manufacturing under USP 823 or CFR 212 guidelines
	-Dosimetry studies in rodents	-Dosimetry studies in rodents	-Dosimetry studies in rodents
	-Limited safety/tox studies in rodents (determined by institutional RDRC)	-Toxicology studies in 1 species	-Toxicology studies in 2 species^a^
		-No safety pharmacology studies	-Safety pharmacology in 2 species^a^
		-No genotoxicity studies	-Genotoxicity studies^a^
Approval	Approval by RDRC and IRB	Approval by FDA and IRB	Approval by FDA and IRB
Subject #	Up to 30	Up to 30	No limit

The purpose, requirements, approval process, and subject numbers for the three application paths available to PET probes are provided in this table

^a^The amount and type of preclinical safety and toxicology studies required to support a PET probe IND are evaluated on a case-by-case basis and can be reduced, if deemed appropriate, by the FDA

1. Radioactive Drug Research Committee (RDRC) is an FDA-sanctioned institutional committee, defined in the Code of Federal Regulations (CFR), Volume 21, Part 361.1 [6–8], that provides a review and approval mechanism for investigators interested in conducting basic human subject research with PET imaging probes. These clinical studies are limited to 30 human subjects, with no diagnostic intent as part of a phase 0 trial. Typically, limited preclinical data is required for the approval of RDRC applications, and, as a result, the cost of obtaining approval for conducting these basic research studies under RDRC is relatively low. The following conditions are required for RDRC approval:

(a) The investigation is considered basic science research and is for the purpose of advancing scientific knowledge, (b) the study is approved by an FDA-sanctioned local RDRC, (c) the pharmacologic dose of the probe to be administered is known not to cause any detectable pharmacological effects in humans, and (d) the radiation dose is justified by the quality of the study such that the total amount of radiation to the human subject must be the smallest dose practical to perform the study without compromising the benefits of the study.

2. Exploratory Investigational New Drug (eIND) Application [9] provides an opportunity to conduct first-in-human phase 0 studies of PET imaging probes involving a limited human pharmacological dose, defined as equal to or less than 100 μg for imaging agents and 30 nmol for protein products, with no diagnostic intent. The eIND approach permits lower-cost human imaging studies through reduced requirements for preclinical animal testing compared to the traditional IND approach. The eIND also serves as a useful screening approach by allowing the sponsor to evaluate two to five PET probes simultaneously under one application and select the top candidate for further clinical development. After eIND studies are completed, the eIND must be withdrawn and further studies with the imaging probe are to be conducted under a traditional IND. This screening approach was utilized successfully as part of the regulatory process for AMYvID [10].

If a PET probe study does not qualify for approval through the RDRC or eIND mechanisms, or IND exemption, which allows the investigator to conduct a study without submitting an IND if specific criteria are met, the IND approach is the only viable option for introducing the PET probe into human subjects. An IND exemption applies when a lawfully marketed PET probe investigation (a) is not intended to support FDA approval of a new indication, labeling change, or advertising change; (b) does not involve a change in route of administration, dosage level, or use in a patient population or other factors that significantly increase the risk to subjects; (c) is conducted in compliance with the Institutional Review Board (IRB), defined in 21 CFR Part 56, informed consent, defined in 21 CFR Part 50 [11], and promotion of investigational drugs, defined in 21 CFR 312.7 [12].

3. IND Application, defined in 21 CFR Part 312 [13, 14], is the mechanism for introducing promising investigational PET probes into phase 1 clinical trials and further clinical development in phases 2 and 3 for obtaining efficacy and additional human safety data in support of a New Drug Application (NDA) approval. The FDA IND guidelines require Good Laboratory Practice (GLP) compliant [15] preclinical data including safety pharmacology and toxicology studies in two species, one in rodent and one in nonrodent. In addition, rodent dosimetry data is required in support of the IND application. The amount of preclinical safety and toxicology studies for PET probes administered in trace mass doses, which are generally considered to be below pharmacologic doses and many orders of magnitude below toxic effects, are evaluated on a case-by-case basis and can be reduced, if deemed appropriate, by the FDA during the pre-IND meetings.

## Approach

**Fig. 2.**
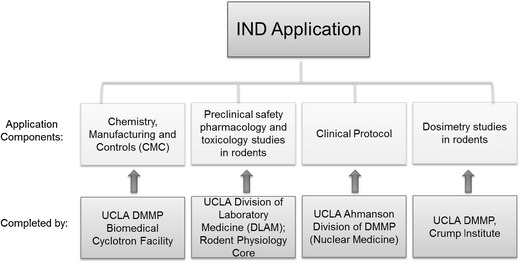
PET probe IND application components. Chemistry, Manufacturing, and Controls (CMC) procedures and data, preclinical safety pharmacology and toxicology data, dosimetry data, and a clinical protocol are required to be submitted to the FDA as components of a PET probe IND application. At UCLA, the data and protocols in support of these components have been obtained from the Biomedical Cyclotron Facility, DLAM, Rodent Physiology Core, Crump Institute for Molecular Imaging and the Ahmanson Translational Imaging Division in DMMP that contains the Nuclear Medicine clinic.

In the past decades, the UCLA Department of Molecular and Medical Pharmacology (DMMP) and its Ahmanson Translational Imaging Division that contains the Nuclear Medicine and PET/CT research and clinical service have utilized and continue to utilize the local RDRC approval mechanism for basic human research on PET probes. Although RDRC will continue to be a path for academic scientists to obtain valuable human bio-distribution data for the purpose of basic research, first-in-human studies cannot be conducted under RDRC. In addition, the FDA Modernization Act requires investigational use of a PET probe to be conducted under an IND, unless IND exemption is granted. To satisfy these requirements and transition promising PET probe candidates from the preclinical stage to clinical research, in compliance with the FDA Modernization Act, we have recently developed an economical and streamlined method to obtain INDs. This approach has been possible through a collaborative effort among several professional groups at UCLA to obtain data and establish protocols that meet the following critical components of IND applications (Fig. 2):

- Preclinical toxicology and safety pharmacology studies are performed by the UCLA Division of Laboratory Animal Medicine (DLAM). This testing facility is maintained under GLP [15] and through a collaborative effort with the Rodent Physiology Core in the Department of Physiology at UCLA.
- Preclinical bio-distribution and dosimetry studies are conducted by the UCLA Crump Institute for Molecular Imaging of DMMP. These studies evaluate the bio-distribution of each probe in three mice and calculate dosimetry in each organ using the FDA-approved OLINDA software [16]. In addition to the discovery and technology inventions for *in vitro* and *in vivo* preclinical molecular imaging diagnostics, the Crump Preclinical Imaging Technology Center facilitates *in vivo* preclinical imaging studies for faculty, students, and their collaborators.
- Chemistry, Manufacturing, and Controls (CMC) are developed and performed by the Biomedical Cyclotron facility in the Ahmanson Translational Imaging Division of DMMP compliant with cGMP requirements (CFR 212 compliant for approved PET probes and USP Chapter <823 > compliant for investigational PET probes). The Biomedical Cyclotron is involved in the development and production of PET probes for research and clinical service within the UCLA David Geffen School of Medicine and health system, as well as conducting research to develop new radiolabelling techniques, probes, and radiosynthesis platforms.
- Clinical protocols and clinical studies are developed and conducted by the UCLA PET/CT facilities in the Ahmanson Translational Imaging Division of DMMP.
- The IND submission strategy was developed through collaboration with our consultant at Perceptive Informatics/PAREXEL.

This collaborative approach has been successfully used to obtain three INDs for the [^18^ F]fluoro-arabinofuranosylcytosine (FAC) analogs within a 1-year time span, as described below.

## FAC Background

**Fig. 3.**
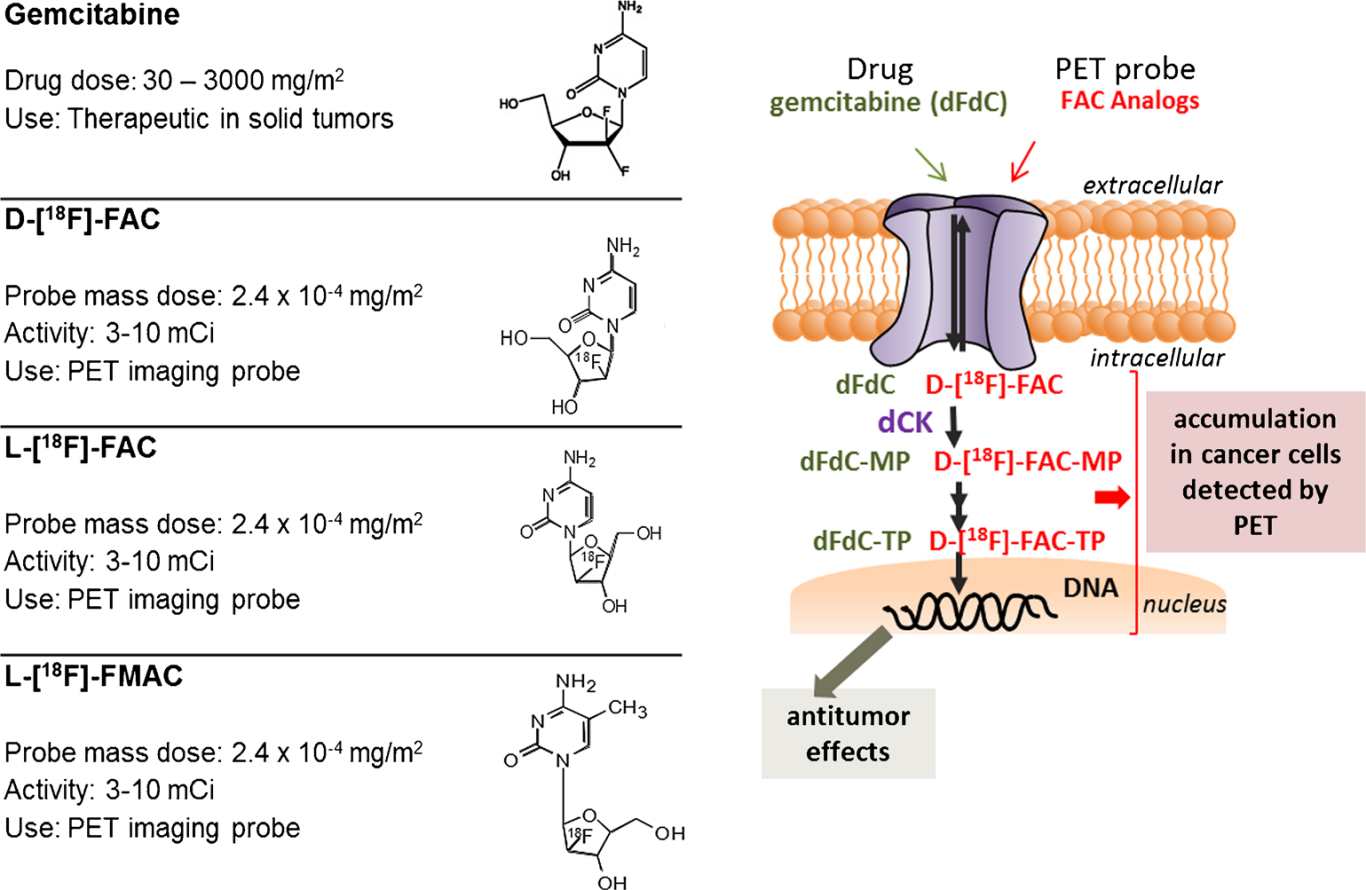
FAC PET probe analogs and gemcitabine. The FAC tracer dose used in PET studies is 2.4 × 10^−5^–2.4 × 10^−7^ times the therapeutic dose of gemcitabine [23]. FAC and gemcitabine follow the same transport and biochemical pathway in normal and cancer cells and are activated through phosphorylation by the enzyme dCK. Reprinted with permission of Springer [24].

Deoxycytidine kinase (dCK) [17] is a rate-limiting enzyme in the deoxyribonucleoside salvage pathway which is expressed predominantly in rapidly proliferating cells, including hematopoietic progenitors in thymus and bone marrow, activated lymphocytes, and cancer cells [18]. dCK is therapeutically important because it phosphorylates the 5′-hydroxyl group in a broad range of nucleoside analog pro-drugs, such as gemcitabine, thus converting them to their corresponding pharmacologically active form. FAC analogs, which include 1-(2′-deoxy-2′-[^18^ F] fluoro-β-d-arabinofuranosyl)cytosine (d-[^18^ F] FAC), 1-(2′-deoxy-2′-[^18^ F] fluoro-β-l-arabinofuranosyl)cytosine (l-[^18^ F] FAC), and 1-(2′-deoxy-2′-[^18^ F] fluoro-β-l-arabinofuranosyl)-5-methylcytosine (l-[^18^ F] FMAC), have been designed to closely resemble the structure of the natural dCK substrate, deoxycytidine, and the nucleoside pro-drug gemcitabine [17, 19]. These probes are transported into tissue by the same facilitated transport system used by deoxycytidine and gemcitabine and are competitively phosphorylated by dCK (Fig. 3).

*In vivo* experiments in mice [20] provide evidence that FAC probes accumulate in tissues proportional to the level of dCK activity due to phosphorylation by the enzyme. As a result, the probe accumulation as measured by PET provides the means to assay the dCK enzyme activity *in vivo*, analogous to the use of 2-deoxy-2-[^18^ F]fluoro-d-glucose (FDG) for assay of transport and phosphorylation of glucose [21].

## FAC IND

We utilized the IND approach for investigating the three FAC analogs in human subjects to (a) assess the safety profile of the FAC probes in human subjects and (b) determine the bio-distribution of the three FAC analogs in cancer patients and evaluate whether the *in vivo* assay of dCK enzyme activity with PET in cancer patients correlated with the *in vitro* assays of dCK expression and enzyme activity from surgically excised cancer tissues.

**Fig. 4.**
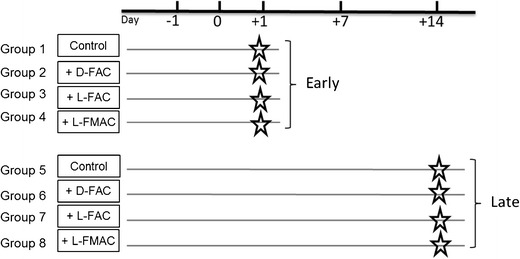
Preclinical study overview for FAC. Sprague-Dawley rats were randomly assigned to eight groups of eight (four male and four female rats in each group) to determine the safety pharmacology and toxicity effects upon an intravenous administration of nonradioactive form of each of the FAC family of probes in rats. The rats were monitored prior to the administration of the test substance and up to 14 days following the administration of the test substance (carrier solution or FAC). The early effects of the administered agents were monitored on day (+1) post injection in groups 1–4, and the late effects were monitored on day (+14) post injection in groups 5–8. Rats in groups 1 and 5 were administered with the carrier solution control and those in groups 2/6, groups 3/7, and groups 4/8 were administered with d-[^19^ F]-FAC, l-[^19^ F]-FAC, and l-[^19^ F]-FMAC, respectively. Administration occurred on day 0.

To support the submission of IND applications for d-[^18^ F] FAC, l-[^18^ F] FAC, and l-[^18^ F] FMAC, a 14-day preclinical safety pharmacology and toxicology study was conducted in Sprague-Dawley rats by UCLA DLAM and the Rodent Physiology Core under GLP conditions (Fig. 4). UCLA maintains AAALAC-accredited animal facilities, and all work was conducted under authorization by the UCLA Animal Research Committee (IACUC equivalent). DLAM had previously conducted a similar study, which resulted in an active IND for [^18^ F]-FHBG, a PET reporter probe for imaging herpes simplex virus type 1 thymidine kinase [22]. Sprague-Dawley rats were selected as the rodent test system for our study to accommodate the need for multiple blood draws and ease of direct blood pressure measurement. The rats were randomly assigned to eight groups of eight (four male and four female rats in each group) to determine the safety pharmacology and toxicity effects upon a single, intravenous administration of nonradioactive forms of each FAC analog in rats. The mass dose of each FAC agent used for these toxicity studies was in excess of 100× the maximum estimated mass dose of the FAC tracer that would be injected into a human subject, equivalent to 27.5 μg/kg. The control groups received only the carrier solution.

In this study, the safety pharmacology and toxicology data, in addition to daily clinical observations for overall health, were obtained upon administering each FAC probe to rats. Safety pharmacology, defined as studies that investigate the potential undesirable pharmacodynamic effects of a compound on physiological functions, was evaluated by measurement of body weight, rectal temperature, heart rate and rhythm, blood pressure, pulse oximetry, and respiration rate. Toxicology studies, which assess the toxicity of a compound to an organism in early and late time points, were evaluated by measuring complete blood counts, clinical/blood chemistry, necropsy, and histopathology.

The early effects of the administered agents were monitored on day (+1) postinjection in groups 1–4, and the late effects of the administered probes were monitored on day (+14) post injection in groups 5–8. Rats in groups 1 and 5 were administered the carrier solution as the control group and those in groups 2 and 6, groups 3 and 7, and groups 4 and 8 were administered with the test probes d-[^19^ F] FAC, l-[^19^ F] FAC, and l-[^19^ F] FMAC, respectively. The preclinical safety pharmacology and toxicology results from these studies are provided in the supplemental section of this article. Overall, the results of these safety pharmacology and toxicology studies with d-[^19^ F] FAC, l-[^19^ F] FAC, and l-[^19^ F] FMAC performed at 100× of equivalent human dose provide evidence that these PET probes are safe for administration into human subjects.

Following completion of preclinical studies for the FAC analogs, a pre-IND meeting was scheduled with the FDA to discuss the best approach for submission of the three INDs, one for each FAC probe. Based on the data package submitted and guidance on preclinical studies required to satisfy microdose administration criteria [9], the FDA considered these studies sufficient and granted a waiver for additional genotoxicity, safety pharmacology, and toxicology studies in a second nonrodent species for the initial phase of the clinical studies. It is important to note that preclinical data requirements and waivers satisfying an IND application may vary from one probe to the next and are determined by the FDA on a case-by-case basis.

INDs, in traditional 11-part paper format, per regulations in 21 CFR 312.23, were submitted to the FDA for each FAC analog (d-[^18^ F] FAC, l-[^18^ F] FAC, and l-[^18^ F] FMAC). Upon completion of the 30-day reviews which responded to questions raised by the FDA, UCLA was granted permission to proceed with the phase 1 clinical studies of 20–30 human subjects for each probe.

## Cost

The costs incurred by our department to obtain data in support of the three FAC INDs totaled $140 K or less than $50 K/imaging probe (Table 2). Two factors influenced the reduced cost for these INDs: (1) the combined safety pharmacology and toxicology assessment was conducted for all three PET probes, which resulted in significant savings by allowing one vehicle control group to be used for all three test groups, and (2) the studies were done in-house using UCLA facilities on a nonprofit fee-for-service basis. The cost of outsourcing a similar safety pharmacology, toxicology, and dosimetry was obtained from two PET contract research organizations and estimated at $150 K for one test probe with a vehicle control.

**Table 2:** Cost of obtaining INDs for three FAC PET probe analogs

Performed by	Studies	Cost
UCLA DLAM	Toxicology and safety pharmacology studies for 3 FAC PET probes and 1 control in rats	$120,000
UCLA Rodent Physiology Core		
Crump Institute for Molecular Imaging- DMMP, UCLA	Dosimetry studies in 9 mice (3 mice/probe)	$10,000
Consulting services: PAREXEL	IND strategy and guidance	$10,000
Total cost		$140,000

The cost for obtaining data in support of IND applications for each of the FAC analogs, d-[^18^ F]-FAC, l-[^18^ F]-FAC, and l-[^18^ F]-FMAC, using UCLA in-house resources, is $140,000. As a result, the cost for each application is calculated to be approximately $50,000

In addition to conducting these studies in an economical manner, the team at UCLA was successful at obtaining the IND for l-[^18^ F] FMAC within 6 months of initiating the preclinical studies in support of the applications. d-[^18^ F] FAC and l-[^18^ F] FAC IND applications were submitted subsequently, and the INDs became active within 2 and 7 months of the l-[^18^ F] FMAC IND.

## Discussion and Conclusion

The DMMP at UCLA has utilized in-house resources in its academic setting to establish and implement a path for satisfying FDA regulatory requirements for approval of PET probe INDs in a streamlined and reduced cost approach. This approach has been validated by expeditiously obtaining the FDA phase 1 approval for three PET imaging probes and was made successful through concerted efforts of professionals in the following critical areas: (1) preclinical safety pharmacology and toxicology; (2) preclinical imaging and dosimetry; (3) chemistry, controls, and manufacturing; (4) clinical study design; and (5) an overall IND submission strategy to satisfy the phase 1 IND requirements in an open and cooperative manner with the FDA. Using the FAC analog PET probes developed at UCLA as a case study, we have demonstrated that this overall phase 1 IND approach can successfully be applied to obtain FDA approval of phase 1 INDs in an academic site efficiently and at a reasonable cost.

**Fig. 5.**
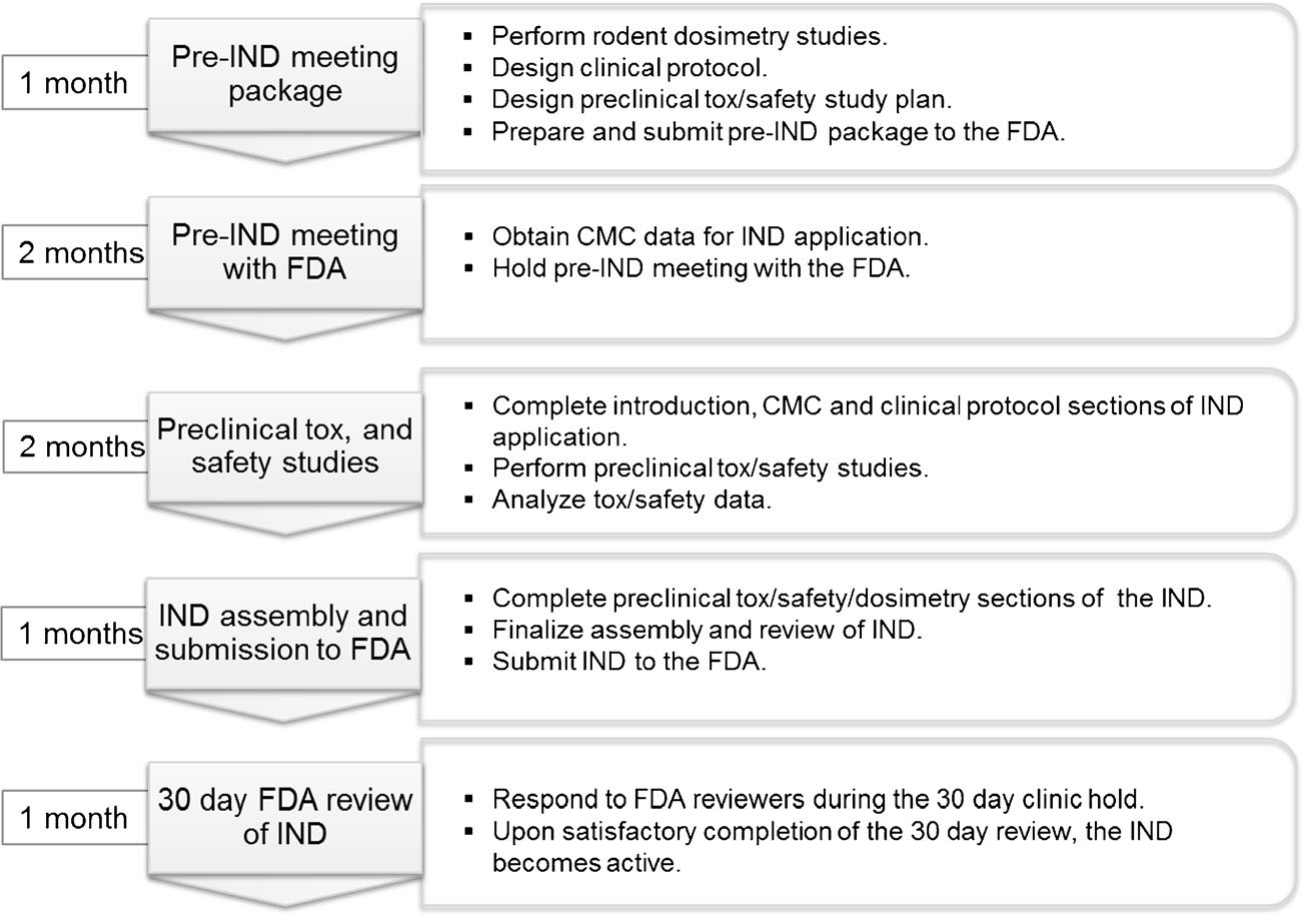
IND submission timeline for PET probes. Based on the UCLA experience, an optimized timeline for generating the data and obtaining a PET probe IND from the FDA is expected to be 7 months. The pre-IND meeting package preparation followed by the pre-IND meeting with the FDA can be completed in 3 months. The pre-IND meeting is not required; however, the meeting is of great value to the sponsor and will allow the FDA and the sponsor to reach an agreement on the types of studies required to support the IND application. The sponsor can conduct the preclinical safety and toxicology studies after the pre-IND meeting parallel to completing the remaining components of the IND application. Completion of the preclinical studies and analysis of the data is expected to last three additional months. At this point, the sponsor will submit the complete IND package to the FDA and the application, if deemed acceptable by the FDA, will become active after 30 days of review.

Although the protocol developed herein has proved successful, we recommend requesting a pre-IND meeting with the FDA prior to initiating preclinical safety pharmacology and toxicology studies in rodents. We surmise that if we had engaged the FDA team earlier and made this request prior to initiating these studies, safety pharmacology studies may have been waived or a limited number of studies would have satisfied the FDA, due to the microdose, tracer levels of PET probes administered according to the eIND guidance document. As such, it is prudent to initiate a pre-IND FDA meeting early in the study development process, which may result in additional cost and time savings for the IND submission. Based on experience gained from the FAC IND project, an optimized project timeline to IND for PET probes is provided here (Fig. 5).

It is the goal of the DMMP at UCLA to continue working closely with the FDA to fulfill the regulatory requirements for PET imaging probes. New requirements outlined in the FDA Safety and Innovation Act (FDASIA) move towards electronic submission of IND applications in Electronic Common Technical Document (eCTD) format. We hope the FDA will make special accommodations for academic sites regarding PET probes in tracer doses by waiving the requirement for submission of eCTD INDs or providing additional tools to academic sites that reduce the cost and time of IND submissions while still maintaining the quality and safety standards relevant to PET probes.

It is critical for academic sites to have a streamlined and cost-effective approach to translate PET imaging probes from preclinical research to clinical investigations and investigate the degree to which preclinical findings for these probes apply to humans. This will allow for the (1) development and introduction of new molecular imaging diagnostics of disease that serve as an array of new assays to investigate the *in vivo* biology of disease in patients, (2) aid in the drug discovery and development processes, and (3) patient stratification for appropriate drug(s) and assessing therapeutic responses.

## Electronic supplementary material

Below is the link to the electronic supplementary material.

ESM 1 (PDF 4693 kb)
