# Supplementary material for: INDs for PET Molecular Imaging Probes—Approach by an Academic Institution
Source: Mol Imaging Biol. 2014 Apr 15;16(4):441–8. doi: 10.1007/s11307-014-0735-2 (PMC4097325; doi:10.1007/s11307-014-0735-2)
Supplement: Supplementary file 1 — (PDF 4693 kb) [file 11307_2014_735_MOESM1_ESM.pdf]

Supplemental Material

INDs for PET molecular imaging probes- approach by an academic institution

Molecular Imaging and Biology

Sherly Mosessian[1], Sandra M. Duarte-Vogel[2], David B. Stout[1,3], Kenneth P. Roos[4], Gregory W. Lawson[2], Maria C. Jordan[4], Amanda Ogden[2], Cheryl Matter[1], Saman Sadeghi[1,5], George Q. Mills[6], Heinrich R. Schelbert[1], Caius G. Radu[1,5], Johannes Czernin[1,5], Marcelo Couto[2], Michael E. Phelps[1,3,5].

[1]Department of Molecular and Medical Pharmacology, David Geffen School of Medicine at University of California Los Angeles, Los Angeles, CA, USA

[2]Division of Laboratory Animal Medicine, David Geffen School of Medicine at University of California Los Angeles, Los Angeles, CA, USA

[3]Crump Institute for Molecular Imaging, David Geffen School of Medicine at University of California Los Angeles, Los Angeles, CA, USA

[4]Department of Physiology, David Geffen School of Medicine at University of California Los Angeles, CA, USA

[5]Ahmanson Translational Imaging Division, David Geffen School of Medicine at University of California Los Angeles, Los Angeles, CA, USA

[6]Perceptive Informatics/Parexel, Gaithersburg, MD, USA

Corresponding author: Sherly Mosessian Ph.D, University of California, Los Angeles, Department of Molecular and Medical Pharmacology, 650 Charles E. Young Dr. South, CHS 23-148, Los Angeles, CA 90095. Phone:(310) 267-1188; Email: smosessian@mednet.ucla.edu

MATERIALS AND METHODS

Study subjects

Adult female (200-220g) and male (300-320g) Crl:CD®SDIGSBR (Sprague-Dawley) rats purchased from Charles River Laboratories, Inc., Wilmington, MA, were used for this study. A total of 64 rats (32 male and 32 female) were assigned to eight groups composed of four male and four female rats in each group. Only rats that appeared healthy were selected for the study. Prior to the beginning of the study, the rats were assigned to one of the eight groups based on random number generation.

Bias was controlled by randomization and by blinding of the study to the technicians who administered the test substance and performed clinical observations of the animals, and to the pathologist evaluating the rats at necropsy and histopathology. The identity of the animal group assignment was not available to the dosing technician, or to the pathologist performing necropsies and histopathology, until the results were collected.

Animal husbandry

Rats were single housed, in a dedicated room, in clear LPI polycarbonate solid bottom microisolator cages; dimensions 19Lx10 1/2Wx8H; floor space: 143 square inches.

Cages with Alpha-dri bedding were changed a minimum of twice per week or if the bedding became excessively wet or soiled, to keep the animals dry and clean. The animal room was independently supplied with at least 10 changes per hour of 100% fresh air. Room temperature was maintained between 71.8-76.8°F, monitored and recorded daily. Room humidity was maintained between 34-60% and was also monitored and recorded daily. An automatically-controlled 12-hour light:12-hour dark fluorescent light cycle was maintained. Each dark period began at 18:00 hrs PST.

Rats were fed ad libitum, Certified Rodent Diet(R) #5002 (LabDiet. PMI Nutrition International). Rats were provided ad libitum RO (Reverse Osmosis) water from individual bottles attached to the cages throughout their stay in the Testing Facility. UCLA maintains AAALAC accredited animal facilities and all work was conducted under authorization by the UCLA Animal Research Committee (IACUC equivalent).

Test substance

Non-radioactive FAC compounds were purchased from Moravek Biochemicals and Radiochemicals (Brea, CA) and dissolved in 1% ethanol in saline. The technician administering the test substance was blinded as to the identity of the animals. A single 100μl volume dose of either the control (only the carrier solution of 1% ethanol in saline) or 27.5μg of non-radioactive FAC per kilogram of body weight was administered intravenously to the rats. This is equivalent to 0 or 100X the maximum tracer mass of FAC administered to human subjects, respectively.

Physiological assessments

Acute arterial blood pressure measurements were obtained via femoral or carotid artery catheterizations to determine the hemodynamic status of the isoflurane anesthetized rats. The Electrocardiogram (ECG) was simultaneously recorded via needle electrodes inserted under the skin in the lead II configuration. These pressure and ECG data were acquired via Grass amplifiers, then digitized, displayed and analyzed in real time with HEM V4.0 software (Notocord Systems, France) on a PC. Heart rates were determined from the R-R interval of the ECG. The oxygen saturation was monitored simultaneously with a MouseOx system and displayed with WinDaq software on the same PC. Baseline data was recorded continuously for at least 15-30 minutes to ensure that physiological levels of pressures, oxygen saturation and heart rates were achieved.

Blood collection

Approximately 1.0-1.5 ml of whole blood were collected by caudal lateral vein bleed under isoflurane anesthesia from rats in groups 1-4 to determine baseline values at the end of the acclimation period (day -1). Subsequent blood samples were collected terminally before pentobarbital overdose euthanasia at day +1 (groups 1-4) and day +14 (groups 5-8) just prior to euthanasia and necropsy, as shown in figure S1. The whole blood samples were divided as follows: at least 350 µL into an EDTA-containing tube (submitted for hematology [CBC, complete blood count]) and 0.5-1.0 mL into a serum-separator tube for blood chemistries. Samples in serum-separator tubes were centrifuged at 2000 x g for 15 minutes after clotting. The serum was evaluated for hemolysis according to colorimetric scale. Hemolysis was acceptable in the range of 20-50, according to the scale. Samples collected for CBC were assessed before running the test for clots. Smears were prepared in the diagnostic lab and read by a pathologist if the CBC equipment (HemoVet) flagged the results.

Euthanasia and necropsy

At scheduled times; blood was collected under general anesthesia using a 24G catheter placed in the lateral caudal vein. Using the same intravenous catheter, animals were euthanized with 100mg/kg pentobarbital. Immediately after euthanasia necropsies were conducted by a pathologist.

Gross necropsy and histopathology

Each rat was weighed on a calibrated, digital scale immediately upon euthanasia and before necropsy. A complete necropsy was performed on each rat and all gross lesions were recorded. Additionally, the following organs were weighed and recorded for comparison between the treated and non-treated groups: brain, heart, thymus, liver, spleen, right and left kidney, and testes when applicable. Samples of all of the organs were collected for histologic examination. All tissues were immersed in 10% formalin immediately after collection. The tissues were sectioned for paraffin embedding within 24-48 hours post fixation. The UCLA-DLAM staff

veterinary pathologist performed or directly supervised all tissue sectioning. The staff veterinary pathologist performed all histopathologic evaluations for this study.

Gross necropsy included an initial physical examination of external surfaces and all orifices. A skin incision was made extending from the mandible through the anogenital region. The incision was extended through the subcutaneous tissues and muscular layers. An internal examination of tissues and organs in situ was performed. The following was examined: external and internal portions of all hollow organs; the external surfaces of the brain and spinal column, the nasal cavity and neck with associated organs and tissues; the thoracic, abdominal and pelvic cavities with associated organs and tissues; and the musculoskeletal carcass. The lungs were perfused with neutral buffered 10% formalin. The entire carcass was fixed in 10% formalin post necropsy and filed for future sectioning if needed.

RESULTS

Study plan

Sprague-Dawley rats were randomly assigned to eight groups of eight rats (four male and four female rats in each group) to determine the safety pharmacology and toxicity effects upon one, single, intravenous administration of non-radioactive form of each of the FAC PET probe analogs in rats (**Figure S1**). The rats were monitored prior and up to 14 days following the administration of the test substance (carrier solution or FAC). The early effects of the administered agents were monitored on day (+1) post injection in groups 1-4 and the late effects of the administered agents were monitored on day (+14) post injection in groups 5-8. Rats in groups 1/5 were administered with the carrier solution control and those in groups 2/6, groups 3/7 and groups 4/8 were administered with the test agents D-[^{19}F]-FAC, L-[^{19}F]-FAC and L-[^{19}F]-FMAC respectively.

The dose of each FAC agent used for these toxicity studies was in excess of 100x the maximum estimated dose of the FAC tracer that may be injected into a human subject, equivalent to 27.5ug per kg. The control groups received the carrier solution instead.

Safety pharmacology

The safety pharmacology parameters evaluated during the study included, body weight, body temperature, heart rate, blood pressure, blood oxygen levels, respiration rate and daily clinical observations.

Body weight: Rats were purchased from Charles River Laboratories with average weights of 200-220grams and 300-320grams for female and male rats respectively. Body weight measurements were taken on days -1, 0, +1, +7 and +14 and averaged for males and females in vehicle control (groups 1 and 5), D-FAC (groups 2 and 6), L-FAC (groups 3 and 7) and L-FMAC (groups 4 and 8) administered rats. The results demonstrated no significant body weight

difference in D-FAC, L-FAC, L-FMAC male or female rats compared to the control groups along the course of the study (**Figure S2 A, B and C**).

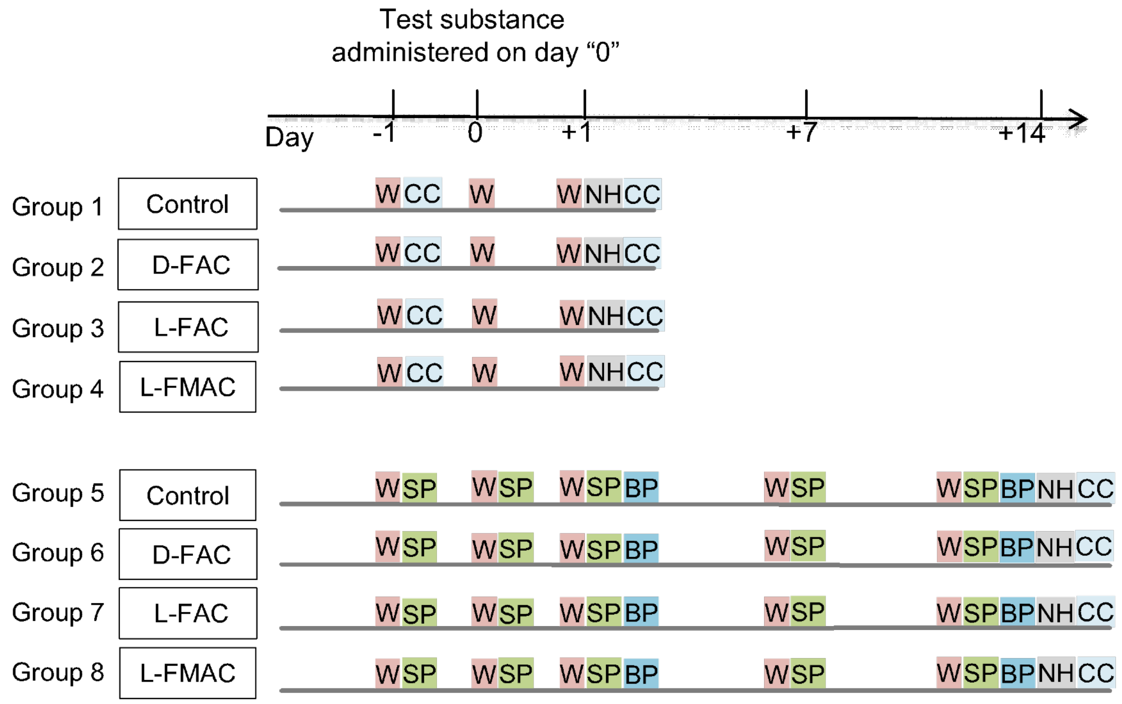

Figure S1. Preclinical study plan. Sprague-Dawley rats were randomly assigned to eight groups of eight rats (four male and four female rats in each group) to determine the safety pharmacology and toxicity effects upon one, single, intravenous administration of non-radioactive form of each of the FAC PET probe analogs. Rats in groups 1/5 were administered with the carrier solution control and those in groups 2/6, groups 3/7 and groups 4/8 were administered with the test agents D-[^{19}F]-FAC, L-[^{19}F]-FAC and L-[^{19}F]-FMAC respectively. Groups 1-4 were monitored on day +1 and groups 5-8 were monitored on day +14. Injection occurred on day 0. Definition for abbreviations: W- Weight, CC- Clinical Chemistry, NH- Necropsy and Histopathology, SP- Safety Pharmacology, BP-Direct Blood Pressure measurement.

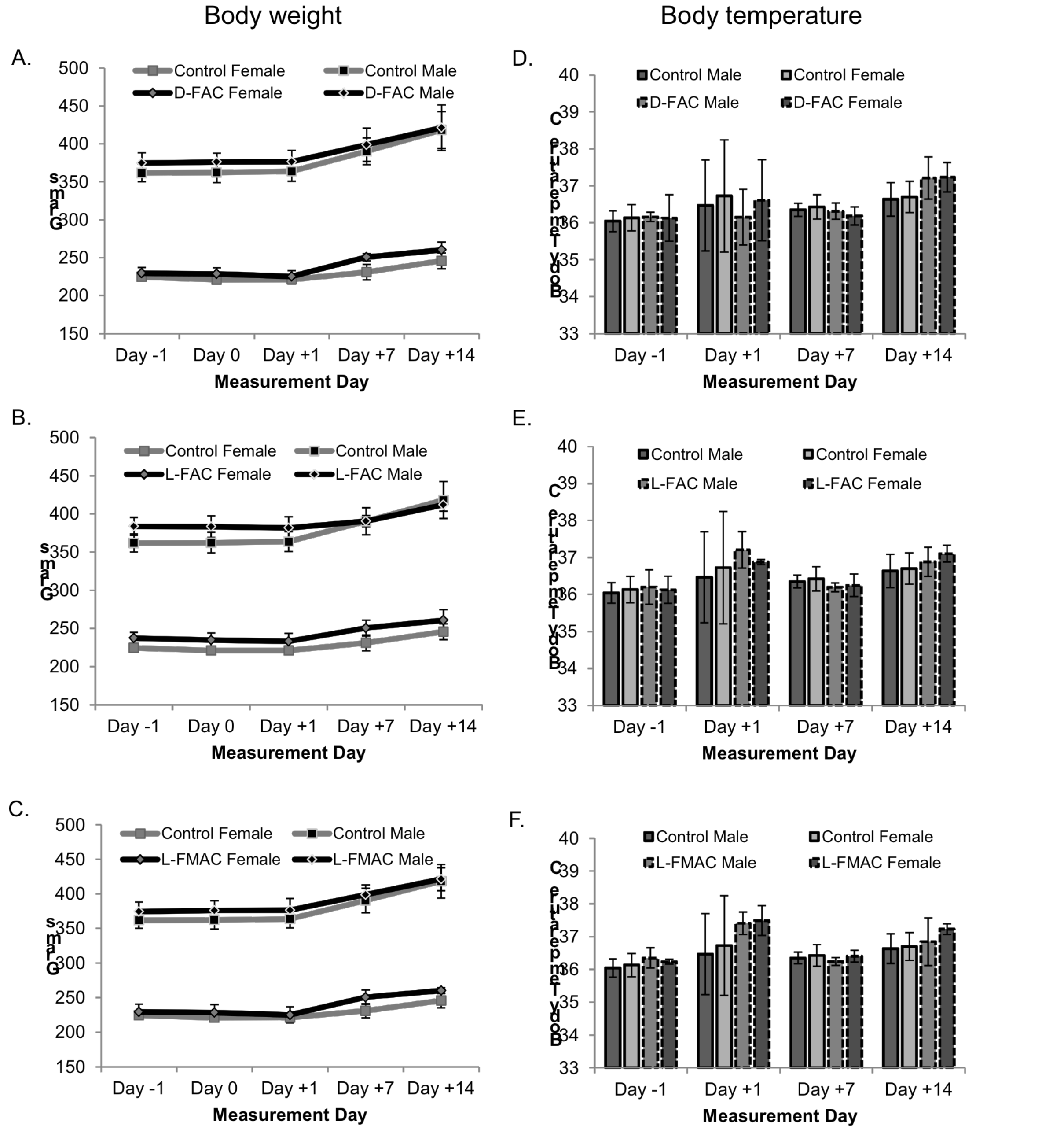

Figure S2. Body weight and temperature measurements. (**A-C**) Body weight measurements were taken on days -1, 0, +1, +7 and +14 and averaged for males and females in vehicle control, D-FAC (**A**), L-FAC (**B**) and L-FMAC (**C**) administered rats. (**D-E**) Rectal body temperatures were recorded on days -1, +1, +7 and +14 for males and females in the vehicle control, D-FAC (**D**), L-FAC (**E**) and L-FMAC (**F**) administered rats. Standard deviations were calculated and incorporated in the graphs.

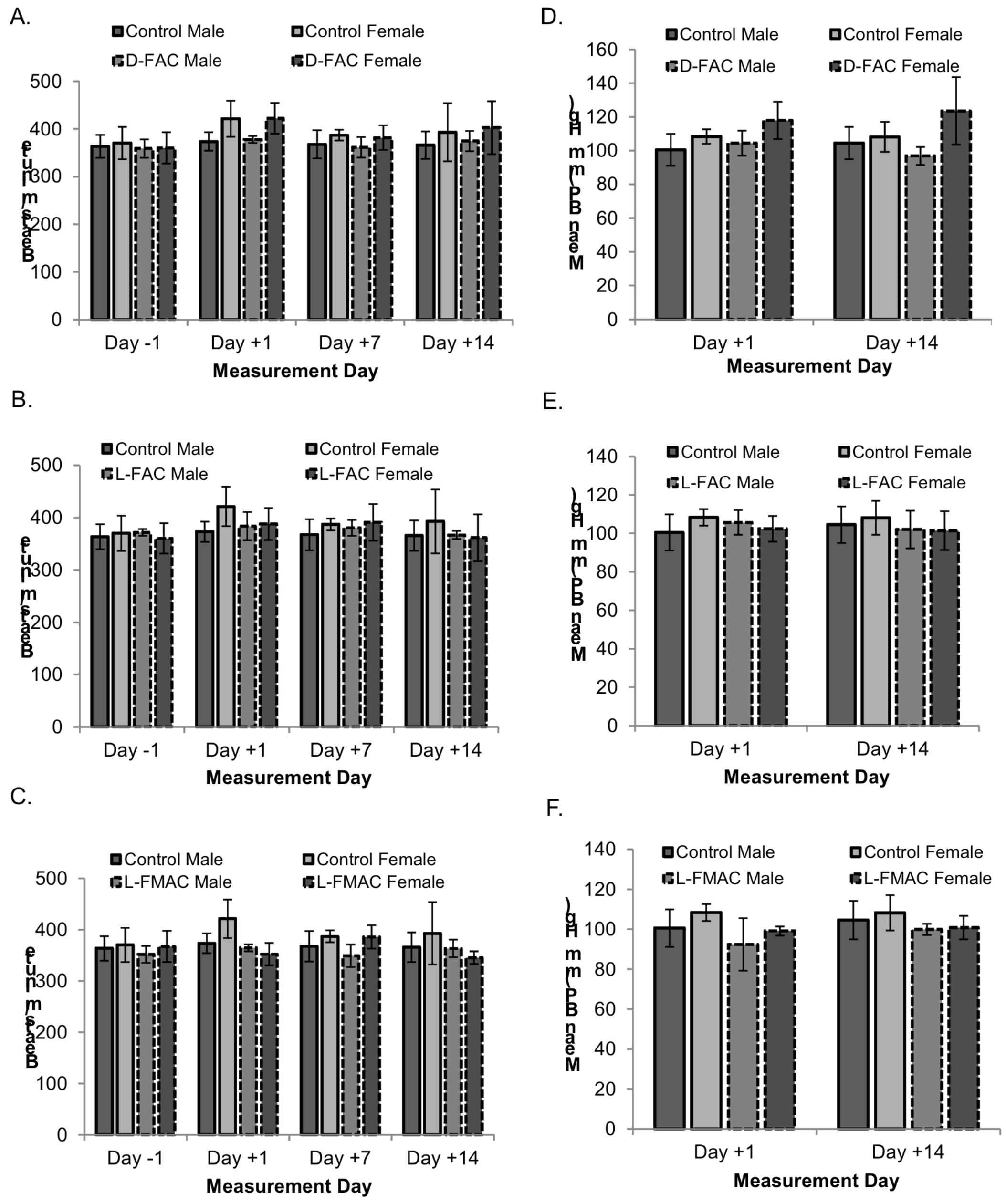

Figure S3. Heart rate and mean blood pressure measurements. (A-C) Electrocardiogram (ECG) traces were recorded on days -1, +1, +7 and +14 for males and females in the vehicle control, D-FAC (**A**), L-FAC (**B**) and L-FMAC (**C**) administered rats. **(D-F)** Mean blood pressure measurements, from the femoral artery, were obtained on days +1 and +14 from males and females for vehicle control, D-FAC (**D**), L-FAC (**E**) and L-FMAC (**F**) administered rats.

Body temperature: Rectal body temperatures were recorded on days -1, +1, +7 and +14 for males and females in the vehicle control (group 5), D-FAC (group 6), L-FAC (group 7) and L-FMAC (group 8) administered rats. The average body temperature results ranged during the course of the study as follows: 36.0-36.7 (Vehicle Control), 36.2-37.2 (D-FAC), 36.1-37.2 (L-FAC) and 36.2-37.5 (L-FMAC). The standard deviation calculations demonstrated no significant difference in body temperatures in D-FAC, L-FAC, L-FMAC male or female rates compared to the control groups (**Figure S2 D, E and F**).

Cardiovascular parameters: ECG traces were recorded on days -1, +1, +7 and +14 for males and females in the vehicle control (group 5), D-FAC (group 6), L-FAC (group 7) and L-FMAC (group 8) administered rats. Heart rate measurements ranged during the course of the study as follows: 364-421 beats per minute (bmp) (Vehicle Control), 360-422 bpm (D-FAC), 360-388 bpm (L-FAC) and 352-386 bpm (L-FMAC). The standard deviation calculations and ECG traces demonstrated that the control and test article injected male and female rats displayed normal heart rates (**Figure S3 A, B, and C**), ECG waves and rhythm.

Blood pressure (BP): Direct BP measurements, from the femoral artery were obtained on days +1 and +14 from males and females in groups 5-8. Mean BP (**Figure S3 D, E and F**), Systolic BP (**Figure S4 A, B and C**) and Diastolic BP (**Figure S4 D, E and F**) measurements were calculated and displayed no significant differences between control and test article administered rats on days +1 and +14.

Pulse oximetry and respiration rate: Hemoglobin oxygen saturation levels and respiration rates for groups 5-8 were obtained on days -1, +1, +7 and +14. The average percent blood oxygen level of all the rats were 99-100% and considered normal (**Figure S5 A, B and C**). No significant changes were observed in the respiration rates (**Figure S5 D, E and F**) between the control and the test article administered groups.

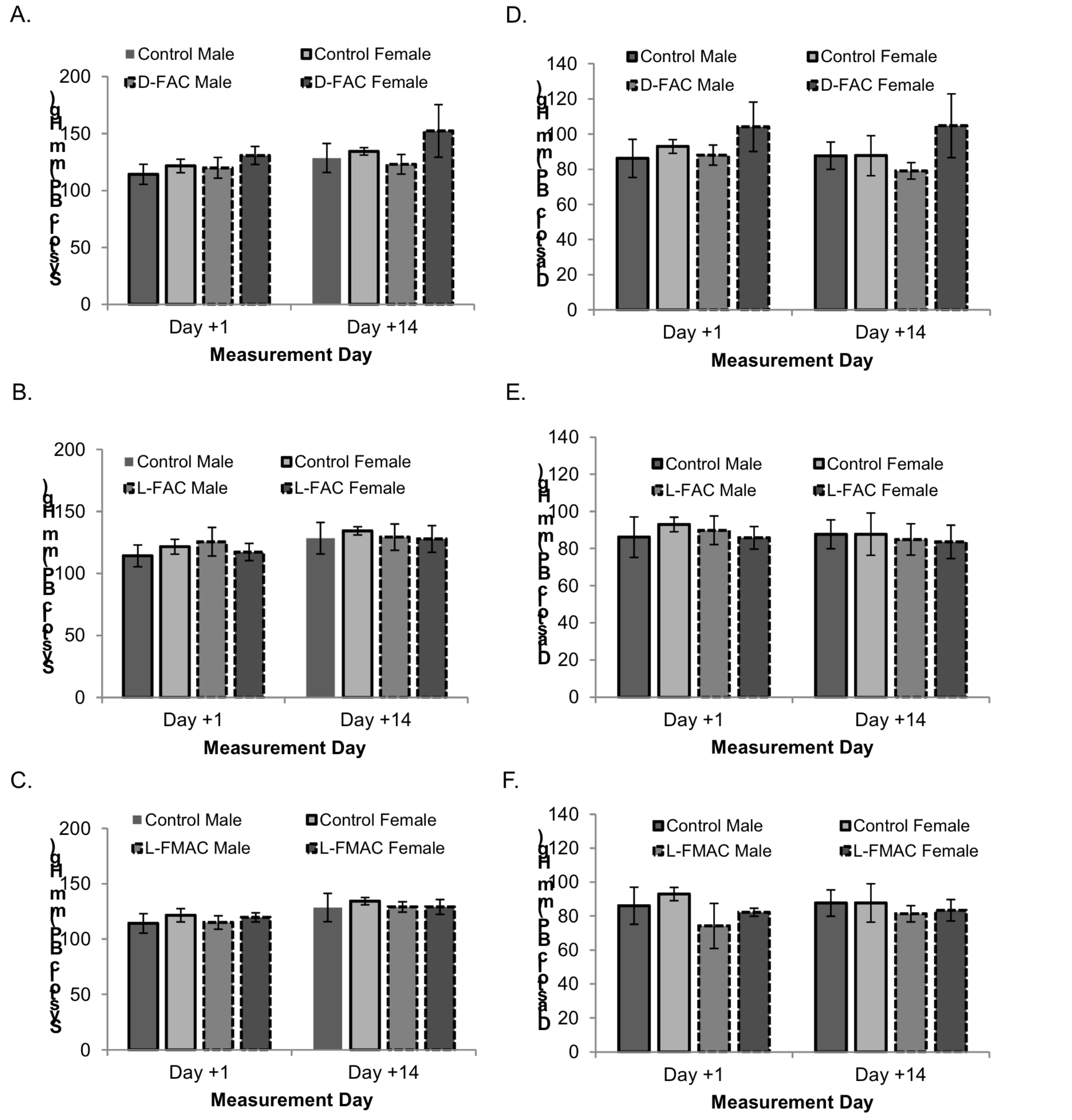

Figure S4. Systolic and diastolic blood pressure measurements. Systolic (**A-C**) and diastolic (**D-E**) blood pressure measurements, from the femoral artery, were obtained on days +1 and +14 from males and females for vehicle control, D-FAC (**A** and **D**), L-FAC (**C** and **E**) and L-FMAC (**D** and **F**) administered rats.

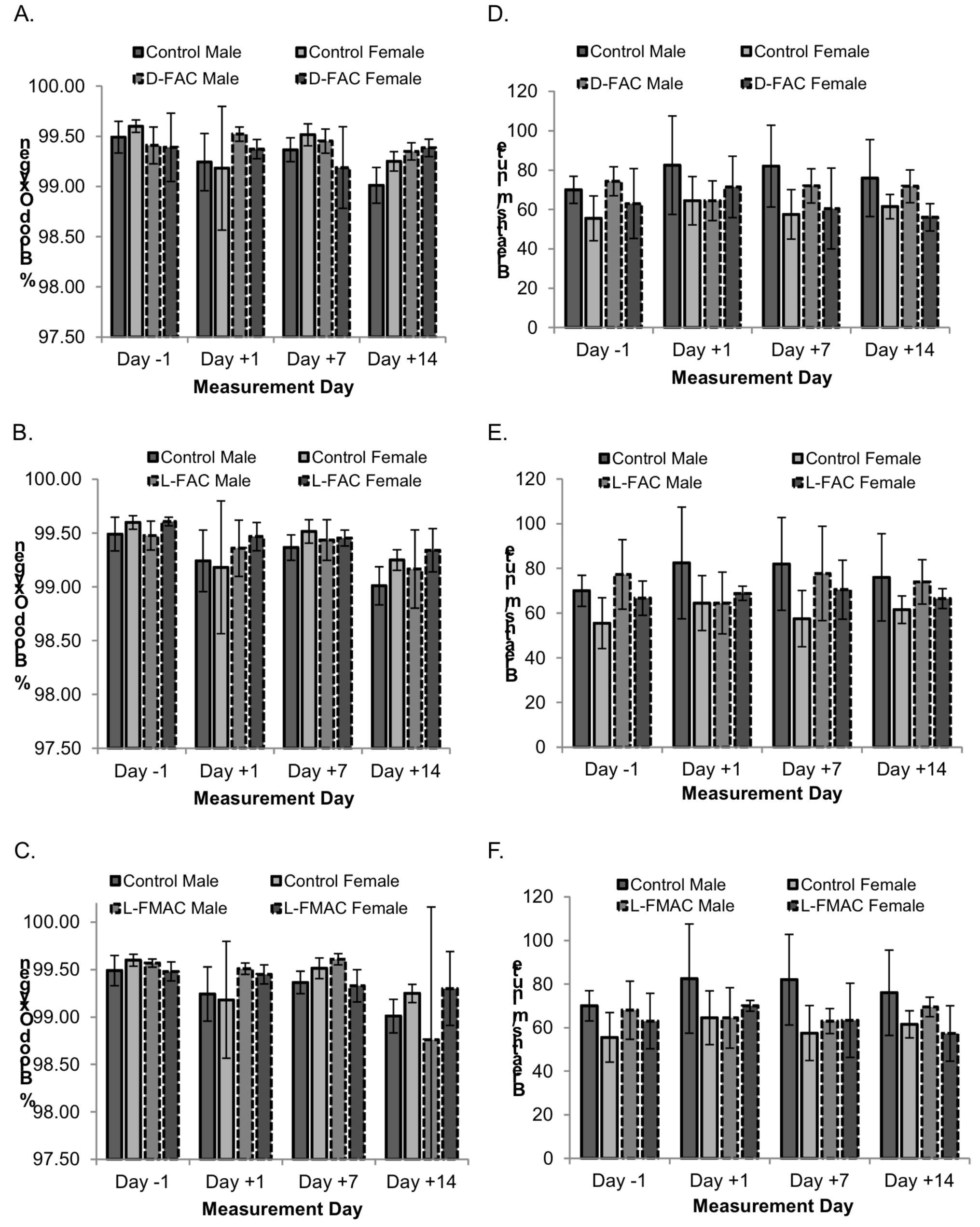

Figure S5. Pulse oximetry and respiration rate measurements. Hemoglobin oxygen saturation levels **(A-C)** and respiration rates **(D-F)** were obtained on days -1, +1, +7 and +14 from males and females for vehicle control, D-FAC (**A** and **D**), L-FAC (**C** and **E**) and L-FMAC (**D** and **F**) administered rats.

Clinical observations: All the rats involved in the study were monitored daily for clinical signs. No clinical signs linked to the administration of the FAC test substance were observed.

Toxicology studies

The early toxicity (day +1) and late toxicity (day +14) of the agents were determined by obtaining complete blood counts (CBC), clinical chemistry, necropsy and histopathology data in rats from groups 1-4 and 6-8 respectively.

CBC: The CBC results for each group under early (**Table S1A**) and late time points (**Table S1B**) were averaged and the standard deviations displayed. The standard deviations with a $p<0.05$ compared to the same sex controls under respective early or late time points are annotated with an asterisk and highlighted.

Serum chemistry: The serum chemistry results for each group under early (**Table S2A**) and late time points (**Table S2B**) were averaged and the standard deviations displayed. The standard deviations with a $p<0.05$ compared to the same sex controls under respective early or late time points are annotated with an asterisk and highlighted.

Necropsy: The necropsy data results for each group under early (**Table S3A**) and late time points (**Table S3B**) were averaged and the standard deviations displayed. The standard deviations with a $p<0.05$ compared to the same sex controls under respective early or late time points are annotated with an asterisk and highlighted.

A significant change was detected in the heart weight ($P<0.05$) of male and female D-FAC treated groups. Once the data had been presented to the FDA, we were requested to conduct a second toxicity study for the D-FAC group. The heart weight measurements from 6 male and female rats were obtained and the results are displayed in tables S3A and S3B under "Heart Wt 2". As shown, the expanded toxicity study revealed that no changes were observed in the heart weight for the D-FAC administered group compared to its respective control.

Histopathology: Histopathology analysis was performed on tissues obtained from all rats after necropsy on days +1 and +14. The following tissues were examined histologically in addition to any gross lesions: Salivary glands, peripheral and mesenteric lymph nodes, brain (brainstem, cerebellum, mid cerebrum and olfactory), pancreas, thyroid, trachea, esophagus, thymus, heart, lung, kidney, liver, spleen, stomach, duodenum, jejunum, ileum, cecum, colon, reproductive tract (ovary, uterus; testis, seminal vesicles, prostate), bladder, haired skin and skeletal muscle (quadriceps). No significant changes in histopathology findings were observed in the FAC injected group compared to the control.

A.	Group 1		Group 2		Group 3		Group 4	
	Control early		D-FAC early		L-FAC early		L-FMAC early	
Male	Mean	SD	Mean	SD	Mean	SD	Mean	SD
WBC (Thousands/uL)	14.100	3.389	12.167	1.784	17.340	5.358	17.830	2.849
Neutrophil (#)	5.090	1.048	5.127	1.011	5.383	1.770	6.203	1.204
Neutrophil (%)	36.620	4.827	41.923	1.999	30.985	1.602	34.895	4.730
Lymphocytes (#)	8.330	2.454	6.287	0.696	11.013	3.300	10.795	2.092
Lymphocytes (%)	58.528	5.770	51.893	3.720	63.625	2.109	60.488	4.863
Monocytes (#)	0.660	0.228	0.743	0.326	0.893	0.326	0.800	0.291
Monocytes (%)	4.703	1.488	6.053	2.640	5.120	0.867	4.465	1.491
Eosinophils (#)	0.023	0.019	0.013	0.015	0.043	0.033	0.015	0.013
Eosinophils (%)	0.133	0.088	0.100	0.125	0.220	0.146	0.095	0.095
Basophils (#)	0.003	0.005	0.003	0.006	0.010	0.008	0.010	0.000*
Basophils (%)	0.023	0.021	0.030	0.036	0.055	0.052	0.058	0.028
RBC (Million/uL)	7.148	0.892	6.983	0.301	6.623	0.447	7.260	0.237
HB (g/dL)	14.150	1.162	14.567	0.737	12.825	0.506	14.550	0.493
HCT (%)	43.400	3.184	44.467	0.764	43.125	3.097	46.000	1.268
PLT (Thousands/uL)	892.0	132.7	847.7	163.4	754.8	69.2	845.5	23.1
MCV (fL)	61.075	4.291	63.733	2.419	65.100	0.702	63.375	2.520
MCH (pg)	19.900	0.883	20.867	0.416	19.400	1.052	20.025	0.465
MCHC (g/dL)	32.600	1.249	32.800	1.744	29.825	1.803*	31.625	0.670
RDW (%)	15.775	0.457	15.433	0.306	14.975	0.395*	15.700	0.804
MPV (fL)	5.800	0.294	5.733	0.351	5.525	0.250	5.650	0.300
PDW (%)	20.850	1.348	18.800	1.054	19.175	0.624	20.125	0.538

Female	Mean	SD	Mean	SD	Mean	SD	Mean	SD
WBC (Thousands/uL)	12.380	0.885	11.535	1.870	13.750	0.1717*	16.500	3.159*
Neutrophil (#)	4.640	1.587	3.728	0.995	3.878	1.163	4.260	0.486
Neutrophil (%)	37.065	10.198	31.995	5.028	28.220	8.621	27.210	9.751
Lymphocytes (#)	7.143	0.959	7.308	1.078	9.208	1.333*	11.465	3.460
Lymphocytes (%)	58.098	9.908	63.660	6.055	66.940	9.489	68.108	9.569
Monocytes (#)	0.578	0.113	0.485	0.159	0.650	0.250	0.745	0.175
Monocytes (%)	4.670	0.804	4.215	1.464	4.708	1.820	4.490	0.321
Eosinophils (#)	0.018	0.010	0.013	0.005	0.013	0.013	0.020	0.022
Eosinophils (%)	0.145	0.068	0.113	0.038	0.108	0.082	0.115	0.110
Basophils (#)	0.000	0.000	0.000	0.000	0.003	0.005	0.015	0.006*
Basophils (%)	0.020	0.008	0.018	0.013	0.023	0.039	0.080	0.0383*
RBC (Million/uL)	6.765	0.111	6.160	0.361*	6.520	0.316	6.415	0.1473*
HB (g/dL)	13.675	0.171	12.850	0.387*	12.450	0.625*	13.400	0.327
HCT (%)	42.525	1.898	40.500	1.753	41.925	2.027	41.775	2.201
PLT (Thousands/uL)	1004.500	183.297	790.500	184.385	818.750	60.191	736.250	139.471
MCV (fL)	62.925	3.762	65.825	2.442	64.325	0.665	65.150	1.982
MCH (pg)	20.225	0.369	20.900	1.364	19.075	0.15*	20.875	0.793
MCHC (g/dL)	32.200	1.547	31.750	1.529	29.700	0.356*	32.175	2.086
RDW (%)	14.875	0.263	14.200	0.497	14.775	0.655	14.400	0.000*
MPV (fL)	5.950	0.603	5.525	0.377	5.600	0.400	5.700	0.560
PDW (%)	18.875	1.715	18.950	1.173	18.800	0.726	19.000	0.883

Table S1A. Hematology measurements. The complete blood counts results for each group under early time points (**A**) were averaged and the standard deviations displayed. The standard deviations with a p<0.05 compared to the same sex controls under respective early or late time points are annotated with an asterisk and highlighted.

B.	Group 5 Control late		Group 6 D-FAC late		Group 7 L-FAC late		Group 8 L-FMAC late	
Male	Mean	SD	Mean	SD	Mean	SD	Mean	SD
WBC (Thousands/uL)	16.570	1.921	14.187	4.223	15.145	2.031	14.520	3.454
Neutrophil (#)	3.823	0.655	5.297	2.618	4.678	2.504	4.837	1.384
Neutrophil (%)	23.113	3.247	35.847	10.995	30.008	12.174	33.233	3.5331*
Lymphocytes (#)	11.938	1.642	8.467	1.934	9.758	1.424	9.117	2.220
Lymphocytes (%)	71.980	3.756	61.000	9.792	65.288	11.803	62.713	3.5329*
Monocytes (#)	0.793	0.279	0.400	0.079	0.678	0.156	0.523	0.035
Monocytes (%)	4.800	1.592	2.953	1.050	4.503	1.054	3.747	0.778
Eosinophils (#)	0.018	0.017	0.020	0.010	0.030	0.022	0.033	0.012
Eosinophils (%)	0.103	0.091	0.173	0.153	0.188	0.104	0.267	0.126
Basophils (#)	0.000	0.000	0.003	0.006	0.003	0.005	0.003	0.006
Basophils (%)	0.003	0.005	0.033	0.035	0.010	0.014	0.033	0.0153*
RBC (Million/uL)	7.843	0.649	7.910	0.195	8.243	0.484	8.133	0.291
HB (g/dL)	14.875	1.135	14.833	0.681	15.050	0.507	15.433	0.473
HCT (%)	47.425	2.571	47.967	1.680	49.800	1.117	49.900	2.512
PLT (Thousands/uL)	855.3	87.9	818.3	115.8	818.5	119.8	803.7	22.7
MCV (fL)	60.600	2.245	60.633	1.677	60.525	2.765	61.300	1.015
MCH (pg)	18.975	1.053	18.733	0.493	18.300	0.726	18.967	0.116
MCHC (g/dL)	31.350	1.277	30.900	0.781	30.250	0.806	30.967	0.666
RDW (%)	15.400	1.117	15.533	1.050	15.650	0.444	16.367	0.231
MPV (fL)	5.925	0.206	5.900	0.100	6.000	0.383	5.867	0.503
PDW (%)	18.975	0.814	19.967	1.168	19.900	1.137	19.433	1.234

Female	Mean	SD	Mean	SD	Mean	SD	Mean	SD
WBC (Thousands/uL)	14.310	3.420	10.970	1.354	10.670	3.273	12.695	3.507
Neutrophil (#)	3.460	1.460	2.838	0.771	2.485	0.453	4.828	3.022
Neutrophil (%)	23.568	5.218	26.308	8.684	25.028	8.134	39.583	26.548
Lymphocytes (#)	10.243	1.828	7.693	1.809	7.768	3.020	7.340	4.245
Lymphocytes (%)	72.280	4.778	69.585	10.080	70.913	9.541	56.303	26.138
Monocytes (#)	0.583	0.313	0.410	0.102	0.403	0.239	0.505	0.191
Monocytes (%)	3.975	1.551	3.805	1.241	3.948	2.034	3.928	0.753
Eosinophils (#)	0.023	0.013	0.025	0.024	0.013	0.015	0.020	0.012
Eosinophils (%)	0.173	0.074	0.238	0.196	0.110	0.081	0.138	0.079
Basophils (#)	0.000	0.000	0.008	0.010	0.000	0.000	0.005	0.010
Basophils (%)	0.005	0.006	0.065	0.090	0.003	0.005	0.048	0.069
RBC (Million/uL)	7.268	0.319	8.100	0.431*	7.835	0.380	8.070	0.3232*
HB (g/dL)	13.875	0.591	15.000	0.663*	14.575	0.732	15.375	0.9605*
HCT (%)	44.400	3.080	50.900	1.806*	47.625	2.399	48.775	2.3286*
PLT (Thousands/uL)	903.5	109.2	843.5	29.2	799.3	69.6	883.3	146.4
MCV (fL)	61.050	2.009	62.950	2.275	60.800	1.635	60.450	3.064
MCH (pg)	19.100	0.927	18.550	0.603	18.600	1.023	19.075	1.506
MCHC (g/dL)	31.325	1.588	29.450	0.592	30.650	1.443	31.525	1.187
RDW (%)	14.900	0.548	14.775	0.640	14.750	0.379	15.200	0.673
MPV (fL)	5.750	0.129	5.775	0.299	5.800	0.529	5.775	0.287
PDW (%)	19.050	0.785	17.850	0.705	17.950	0.933	19.800	1.344

Table S1B. Hematology measurements. The complete blood counts results for each group under late time points (**B**) were averaged and the standard deviations displayed. The standard deviations with a $p<0.05$ compared to the same sex controls under respective early or late time points are annotated with an asterisk and highlighted.

A.	Group 1 Control early		Group 2 D-FAC early		Group 3 L-FAC early		Group 4 L-FMAC early	
Male	Mean	SD	Mean	SD	Mean	SD	Mean	SD
TPROT (g/dl)	5.650	0.351	6.100	0.200	5.575	0.287	6.200	0.245*
ALT (U/L)	54.000	16.310	45.667	5.508	43.750	7.089	47.000	11.547
AST (U/L)	79.000	24.752	76.333	3.215	64.750	4.272	86.250	9.912
LDH (U/L)	173.500	118.604	223.333	94.511	134.250	109.837	205.250	40.508
CK (U/L)	264.250	116.783	292.000	20.298	168.750	39.399	272.250	117.179
BUN (mg/dl)	16.250	3.304	13.667	1.155	16.250	1.500	16.750	2.062
CHOL (mg/dl)	58.500	8.888	64.000	17.521	63.250	6.946	71.750	5.7951*
ALP (U/L)	276.250	52.182	234.333	51.481	248.750	45.184	261.000	66.648
TBILI (mg/dl)	0.175	0.050	0.200	0.000	0.225	0.050	0.325	0.0957*
GLU (mg/dl)	213.750	72.853	160.333	18.339	161.500	5.260	179.250	23.172
PHOS (mg/dl)	6.900	0.294	7.567	0.289*	7.225	0.499	7.850	0.802
CA (mg/dl)	10.000	0.432	9.800	0.300	9.675	0.275	10.000	0.258
CO2 (mEq/L)	24.475	1.710	24.800	1.868	22.375	0.320	22.775	0.591
CREA (mg/dl)	0.425	0.050	0.400	0.000	0.325	0.05*	0.375	0.050
DBILI (mg/dl)	0.200	0.000	0.2	0.1	0.275	0.096	0.500	0.1633*
ALB (g/ul)	2.850	0.129	3.033	0.153	2.925	0.206	3.100	0.0816*
GGT (U/L)	1.500	1.000	1.667	0.577	0.750	0.500	1.500	1.000
Sodium (mmol/L)	134.533	3.667	136.967	2.155	136.480	2.630	137.050	3.181
Potassium (mmol/L)	5.777	0.283	6.037	0.578	5.648	0.340	6.058	0.508
Chloride (mmol/L)	98.800	3.483	100.067	2.470	101.230	1.873	100.850	2.559

Female	Mean	SD	Mean	SD	Mean	SD	Mean	SD
TPROT (g/dl)	6.200	0.392	6.000	0.141	5.850	0.311	6.425	0.126
ALT (U/L)	49.250	21.899	40.000	3.830	38.500	4.435	42.250	7.500
AST (U/L)	78.250	26.043	74.500	5.686	66.500	6.137	81.250	8.461
LDH (U/L)	164.750	59.707	248.750	142.738	138.000	71.745	226.750	42.813
CK (U/L)	218.500	33.551	276.000	128.356	155.000	60.194	234.500	66.556
BUN (mg/dl)	13.500	1.915	15.000	2.160	15.250	0.957	15.500	1.732
CHOL (mg/dl)	81.250	12.971	66.750	9.323	74.000	6.633	69.000	8.525
ALP (U/L)	139.250	8.342	168.500	31.268	165.750	46.090	176.000	60.503
TBILI (mg/dl)	0.275	0.150	0.300	0.000	0.175	0.050	0.300	0.000
GLU (mg/dl)	195.250	30.038	205.500	57.911	175.000	27.665	200.000	52.058
PHOS (mg/dl)	6.475	0.457	7.425	0.714	6.475	0.403	7.075	0.427
CA (mg/dl)	10.175	0.206	10.175	0.287	9.800	0.523	10.775	0.699
CO2 (mEq/L)	23.600	0.698	23.550	0.858	22.150	0.971	22.350	1.139
CREA (mg/dl)	0.400	0.000	0.500	0.0816*	0.325	0.05*	0.375	0.050
DBILI (mg/dl)	0.300	0.200	0.375	0.096	0.175	0.050	0.325	0.050
ALB (g/ul)	3.200	0.183	3.100	0.082	3.200	0.294	3.350	0.100
GGT (U/L)	0.750	0.500	2.250	0.957*	2.250	1.258	1.750	1.708
Sodium (mmol/L)	136.930	1.155	137.250	1.529	135.230	2.604	135.480	1.903
Potassium (mmol/L)	5.700	0.099	5.473	0.289	5.228	0.362	5.315	0.423
Chloride (mmol/L)	101.830	1.172	102.600	1.960	100.580	2.300	99.900	1.637

Table S2A. Serum chemistry measurements. The serum chemistry results for each group under early time points (**A**) were averaged and the standard deviations displayed. The standard deviations with a $p<0.05$ compared to the same sex controls under respective early or late time points are annotated with an asterisk and highlighted.

B.

Male	Group 5 Control late		Group 6 D-FAC late		Group 7 L-FAC late		Group 8 L-FMAC late	
	Mean	SD	Mean	SD	Mean	SD	Mean	SD
TPROT (g/dl)	5.550	0.342	5.267	0.379	5.750	0.100	6.175	0.1708*
ALT (U/L)	41.750	3.202	38.000	2.646	41.000	2.944	42.250	4.349
AST (U/L)	66.750	7.932	69.333	8.083	66.500	4.509	64.500	2.381
LDH (U/L)	146.750	60.252	194.000	80.988	204.250	61.446	119.000	24.235
CK (U/L)	189.750	57.308	204.000	54.672	186.500	41.008	161.000	14.765
BUN (mg/dl)	12.500	1.000	14.000	1.732	14.750	1.893	16.000	1.826*
CHOL (mg/dl)	56.000	4.397	50.333	0.577	63.000	13.976	62.250	9.323
ALP (U/L)	206.250	27.536	220.000	66.573	200.250	55.614	249.500	12.369*
TBILI (mg/dl)	0.150	0.058	0.233	0.058	0.250	0.058	0.150	0.058
GLU (mg/dl)	191.250	15.392	174.667	12.583	194.250	19.500	182.250	9.912
PHOS (mg/dl)	7.250	0.526	6.733	0.569	6.400	0.606	7.125	0.275
CA (mg/dl)	9.575	0.050	9.800	0.400	9.575	0.427	10.275	0.1500*
CO2 (mEq/L)	24.300	0.938	22.433	0.289*	25.350	0.480	24.225	1.072
CREA (mg/dl)	0.400	0.000	0.400	0.000	0.400	0.000	0.375	0.050
DBILI (mg/dl)	0.200	0.082	0.367	0.0577*	0.275	0.096	0.250	0.058
ALB (g/ul)	2.750	0.129	2.833	0.115	2.800	0.000	2.975	0.1258*
GGT (U/L)	1.250	0.500	0.667	0.577	2.750	0.957*	3.000	0.000*
Sodium (mmol/L)	130.467	0.833	136.000	2.879*	135.500	4.468	139.180	5.140
Potassium (mmol/L)	5.650	0.671	5.990	0.497	5.373	0.578	5.590	0.262
Chloride (mmol/L)	96.933	0.551	100.533	1.0214*	98.125	2.069	101.380	3.717

Female	Mean	SD	Mean	SD	Mean	SD	Mean	SD
TPROT (g/dl)	5.900	0.082	5.475	0.506	6.225	0.2062*	6.133	0.289
ALT (U/L)	41.500	2.646	40.000	12.247	51.250	4.113*	42.000	4.359
AST (U/L)	63.000	7.394	68.750	17.017	72.000	8.042	68.667	4.042
LDH (U/L)	192.000	97.081	187.250	90.842	174.000	65.218	144.670	107.156
CK (U/L)	207.250	58.858	194.000	30.908	161.000	14.095	166.330	39.514
BUN (mg/dl)	13.250	0.500	15.000	2.582	14.000	4.083	14.667	0.5774*
CHOL (mg/dl)	78.250	8.995	68.500	17.972	73.500	11.790	71.000	12.767
ALP (U/L)	168.250	24.527	118.250	23.486*	118.500	24.83*	158.330	11.719
TBILI (mg/dl)	0.200	0.000	0.200	0.000	0.200	0.082	0.200	0.000
GLU (mg/dl)	181.250	4.646	213.000	35.071	173.500	11.387	176.670	15.308
PHOS (mg/dl)	6.175	0.386	6.625	0.544	6.000	0.497	6.367	0.503
CA (mg/dl)	9.700	0.245	10.125	0.435	10.125	0.670	10.200	0.1732*
CO2 (mEq/L)	23.725	1.106	21.625	1.684	25.300	1.510	25.067	0.322
CREA (mg/dl)	0.400	0.000	0.450	0.058	0.425	0.050	0.400	0.000
DBILI (mg/dl)	0.200	0.000	0.225	0.050	0.225	0.096	0.233	0.058
ALB (g/ul)	3.050	0.100	3.000	0.294	3.200	0.141	3.167	0.058
GGT (U/L)	1.500	1.000	1.500	1.291	2.750	0.957	2.333	1.528
Sodium (mmol/L)	135.500	0.625	132.675	7.424	135.080	2.830	136.270	2.026
Potassium (mmol/L)	4.920	0.281	6.220	1.435	4.878	0.357	5.120	0.571
Chloride (mmol/L)	99.000	0.700	96.650	1.760	97.075	0.885*	98.433	1.286

Table S2B. Serum chemistry measurements. The serum chemistry results for each group under late time points (**B**) were averaged and the standard deviations displayed. The standard deviations with a $p<0.05$ compared to the same sex controls under respective early or late time points are annotated with an asterisk and highlighted.

A.

Male	Group 1 Control early		Group 2 D-FAC early		Group 3 L-FAC early		Group 4 L-FMAC early	
	Mean	SD	Mean	SD	Mean	SD	Mean	SD
Body wt	351.833	29.747	301.943	29.404	390.125	14.463	368.960	20.590
Liver wt	17.860	1.985	13.557	1.83*	17.213	1.381	18.498	1.244
Spleen wt	0.655	0.087	0.687	0.096	1.135	0.507	0.728	0.097
Pancreas wt	0.978	0.177	0.933	0.395	1.455	0.262*	1.093	0.205
Rt. Adrenal wt	0.053	0.005	0.043	0.015	0.045	0.010	0.045	0.006
Lt. Adrenal wt	0.045	0.017	0.047	0.006	0.045	0.010	0.045	0.010
Rt. Kidney wt	1.910	0.534	1.650	0.060	1.803	0.107	1.798	0.317
Lt. Kidney wt	1.870	0.429	1.703	0.218	1.815	0.139	1.820	0.310
Thymus wt	0.615	0.137	0.483	0.101	0.710	0.083	0.593	0.155
Brain wt	2.155	0.120	2.120	0.173	2.070	0.108	2.083	0.091
Heart wt 1	2.045	0.218	1.270	0.32*	1.738	0.522	1.793	0.118
Heart wt 2	1.390	0.200	1.400	0.180	N/A	N/A	N/A	N/A
Rt. Testis wt	2.208	0.217	2.107	0.120	2.175	0.089	2.213	0.075
Lt. Testis wt	2.205	0.150	2.177	0.159	2.190	0.132	2.233	0.200
Seminal vesicle + bladder	2.140	0.119	2.217	0.125	2.398	0.253	2.648	0.477

Female	Mean	SD	Mean	SD	Mean	SD	Mean	SD
Body wt	219.848	6.178	219.950	10.483	225.280	8.619	216.138	12.352
Liver wt	10.850	0.879	8.990	0.99*	9.193	0.358*	10.173	1.312
Spleen wt	0.433	0.056	0.528	0.074	0.485	0.031	0.515	0.047
Pancreas wt	0.888	0.142	0.988	0.294	0.678	0.094	0.735	0.087
Rt. Adrenal wt	0.038	0.005	0.050	0.01*	0.045	0.030	0.048	0.015
Lt. Adrenal wt	0.035	0.006	0.053	0.01*	0.050	0.026	0.050	0.016
Rt. Kidney wt	1.173	0.036	1.075	0.100	1.130	0.063	1.133	0.067
Lt. Kidney wt	1.165	0.105	1.058	0.128	1.110	0.072	1.095	0.111
Thymus wt	0.540	0.127	0.560	0.203	0.553	0.057	0.490	0.114
Brain wt	1.905	0.112	2.000	0.154	1.958	0.112	1.918	0.043
Heart wt 1	1.323	0.146	1.043	0.16*	1.008	0.037*	1.183	0.124
Heart wt 2	0.930	0.200	0.900	0.110	N/A	N/A	N/A	N/A
Uterus, ovarian bladder wt	1.218	0.226	1.185	0.078	1.085	0.047	1.110	0.173

Table S3A. Necropsy- organ weights. The necropsy data results for each group under early time points (**A**) were averaged and the standard deviations displayed. The standard deviations with a $p<0.05$ compared to the same sex controls under respective early or late time points are annotated with an asterisk and highlighted.

B.

Male	Group 5 Control late		Group 6 D-FAC late		Group 7 L-FAC late		Group 8 L-FMAC late	
	Mean	SD	Mean	SD	Mean	SD	Mean	SD
Body wt	417.900	24.876	412.377	28.378	411.750	8.057	421.250	16.681
Liver wt	17.653	1.407	17.963	1.812	15.828	1.917	16.993	0.759
Spleen wt	0.815	0.143	0.733	0.074	0.810	0.106	0.890	0.075
Pancreas wt	1.150	0.176	1.183	0.055	1.160	0.181	1.243	0.052
Rt. Adrenal wt	0.040	0.012	0.040	0.017	0.048	0.010	0.040	0.012
Lt. Adrenal wt	0.038	0.010	0.043	0.025	0.043	0.005	0.035	0.010
Rt. Kidney wt	1.925	0.284	1.880	0.174	1.948	0.142	1.978	0.185
Lt. Kidney wt	1.835	0.295	1.883	0.093	1.880	0.134	1.798	0.156
Thymus wt	0.573	0.113	0.490	0.123	0.500	0.057	0.530	0.062
Brain wt	2.103	0.043	2.037	0.025	2.168	0.133	2.143	0.031
Heart wt 1	1.450	0.181	1.540	0.200	1.488	0.046	1.528	0.138
Heart wt 2	1.478	0.165	1.432	0.216	N/A	N/A	N/A	N/A
Rt. Testis wt	2.213	0.251	2.433	0.101	2.463	0.198	2.533	0.132
Lt. Testis wt	2.308	0.220	2.173	0.431	2.403	0.129	2.430	0.209
Seminal vesicle + bladder	3.145	0.388	2.563	0.100	3.295	0.816	2.933	0.367

Female	Mean	SD	Mean	SD	Mean	SD	Mean	SD
Body wt	244.840	11.040	230.883	10.648	260.045	13.516	259.773	3.440
Liver wt	10.350	1.670	9.490	1.146	11.338	1.986	11.383	1.580
Spleen wt	0.540	0.090	0.445	0.026	0.615	0.079	0.597	0.050
Pancreas wt	0.900	0.190	0.773	0.035	0.908	0.123	0.903	0.095
Rt. Adrenal wt	0.050	0.010	0.043	0.010	0.043	0.010	0.047	0.012
Lt. Adrenal wt	0.050	0.020	0.045	0.006	0.043	0.010	0.050	0.010
Rt. Kidney wt	1.210	0.140	1.113	0.140	1.325	0.114	1.253	0.061
Lt. Kidney wt	1.140	0.140	0.840	0.472	1.208	0.083	1.273	0.070
Thymus wt	0.540	0.130	0.450	0.089	0.545	0.052	0.463	0.091
Brain wt	1.880	0.120	1.970	0.093	1.970	0.032	1.953	0.031
Heart wt 1	1.120	0.150	0.895	0.075*	1.138	0.235	1.150	0.288
Heart wt 2	0.927	0.094	0.895	0.060	N/A	N/A	N/A	N/A
Uterus, ovarian bladder wt	1.500	0.410	1.200	0.298	1.370	0.341	1.413	0.169

Table S3B. Necropsy- organ weights. The necropsy data results for each group under late time points (**B**) were averaged and the standard deviations displayed. The standard deviations with a $p<0.05$ compared to the same sex controls under respective early or late time points are annotated with an asterisk and highlighted.
